# Even hotter hotspot: description of seven new species of many-plumed moths (Lepidoptera, Alucitidae) from Mount Cameroon

**DOI:** 10.3897/zookeys.935.49843

**Published:** 2020-05-21

**Authors:** Peter Ustjuzhanin, Vasily Kovtunovich, Vincent Maicher, Szabolcs Sáfián, Sylvain Delabye, Alexander Streltzov, Robert Tropek

**Affiliations:** 1 Altai State University, Lenina 61, Barnaul, RU-656049, Russia; 2 Biological Institute, Tomsk State University, Lenina Prospekt 36, Tomsk 634050, Russia; 3 Moscow Society of Nature Explorers, Bolshaya Nikitskaya 2, Moscow, RU-125009, Russia; 4 Institute of Entomology, Biology Centre of the Czech Academy of Sciences, Branišovská 31, CZ-37005 České Budějovice, Czech Republic; 5 Faculty of Science, University of South Bohemia, Branišovská 1760, CZ-37005 České Budějovice, Czech Republic; 6 Department of Ecology, Faculty of Science, Charles University, Viničná 7, CZ-12844 Prague, Czech Republic; 7 Institute of Silviculture and Forest Protection, University of Sopron, Bajcsy-Zsilinszky u. 4. H-9945 Sopron, Hungary; 8 Herzen State Pedagogical University of Russia, 48, Moika Emb., Saint-Petersburg, 191186, Russia

**Keywords:** *
Alucita
*, Afrotropics, biodiversity, endemic, insects, microlepidoptera, taxonomy, tropical rainforest

## Abstract

Mount Cameroon, SW Cameroon, has already been described as a unique hotspot of the many-plumed moth (Lepidoptera, Alucitidae), with their local diversity unrivalled in the entire Afrotropics. We confirm its importance with description of seven new species: *Alucita bakweri* Ustjuzhanin & Kovtunovich, **sp. nov.**, *Alucita jana* Ustjuzhanin & Kovtunovich, **sp. nov.**, *Alucita bakingili* Ustjuzhanin & Kovtunovich, **sp. nov.**, *Alucita tatjana* Ustjuzhanin & Kovtunovich, **sp. nov.**, *Alucita zuza* Ustjuzhanin & Kovtunovich, **sp. nov.**, *Alucita deja* Ustjuzhanin & Kovtunovich, **sp. nov.**, and *Alucita bokwango* Ustjuzhanin & Kovtunovich, **sp. nov.** These descriptions have raised the known local diversity of many-plumed moth species on Mount Cameroon to 22, i.e., over a quarter of the known Afrotropical biodiversity of this group. This study also emphasises the great conservation importance of the area.

## Introduction

Mount Cameroon has recently been recognised as a unique biodiversity hotspot for many-plumed moths (Lepidoptera, Alucitidae) (Ustjuzhanin et al. 2018), an easily recognisable family of Lepidoptera because of their characteristic six-lobed wings. As reviewed in Ustjuzhanin et al. (2018), Alucitidae are strongly understudied in the Afrotropics. While several recent faunistic (Maicher et al. 2016; Przybyłowicz et al. 2019; Delabye et al. 2020) and taxonomic (Przybyłowicz 2013; Sáfián and Tropek 2016; Yakovlev and Sáfián 2016; Sáfián et al. 2019) discoveries have shown the Mount Cameroon region as an important but largely understudied locality for lepidopteran biodiversity, its importance for Alucitidae biodiversity in particular remains unrivalled. Of 70 Afrotropical species of many-plumed moths, 32 are known from the Guineo-Congolian forest zone (De Prins and De Prins 2019); 15 of these have been already described or reported from Mount Cameroon (Ustjuzhanin et al. 2018).

In this study, we report on a second part of the material sampled during our long-term study of lepidopteran diversity in rainforests of Mount Cameroon (e.g., Maicher et al. 2018, 2019). Seven species of *Alucita* are described as new for science.

## Materials and methods

### Abbreviations

**CUK** personal collections of P. Ustjuzhanin and V. Kovtunovich, Novosibirsk and Moscow, Russia.

**NECJU**Nature Education Centre, Jagiellonian University, Kraków, Poland.

### Material sampling and processing

**Figure 1.**
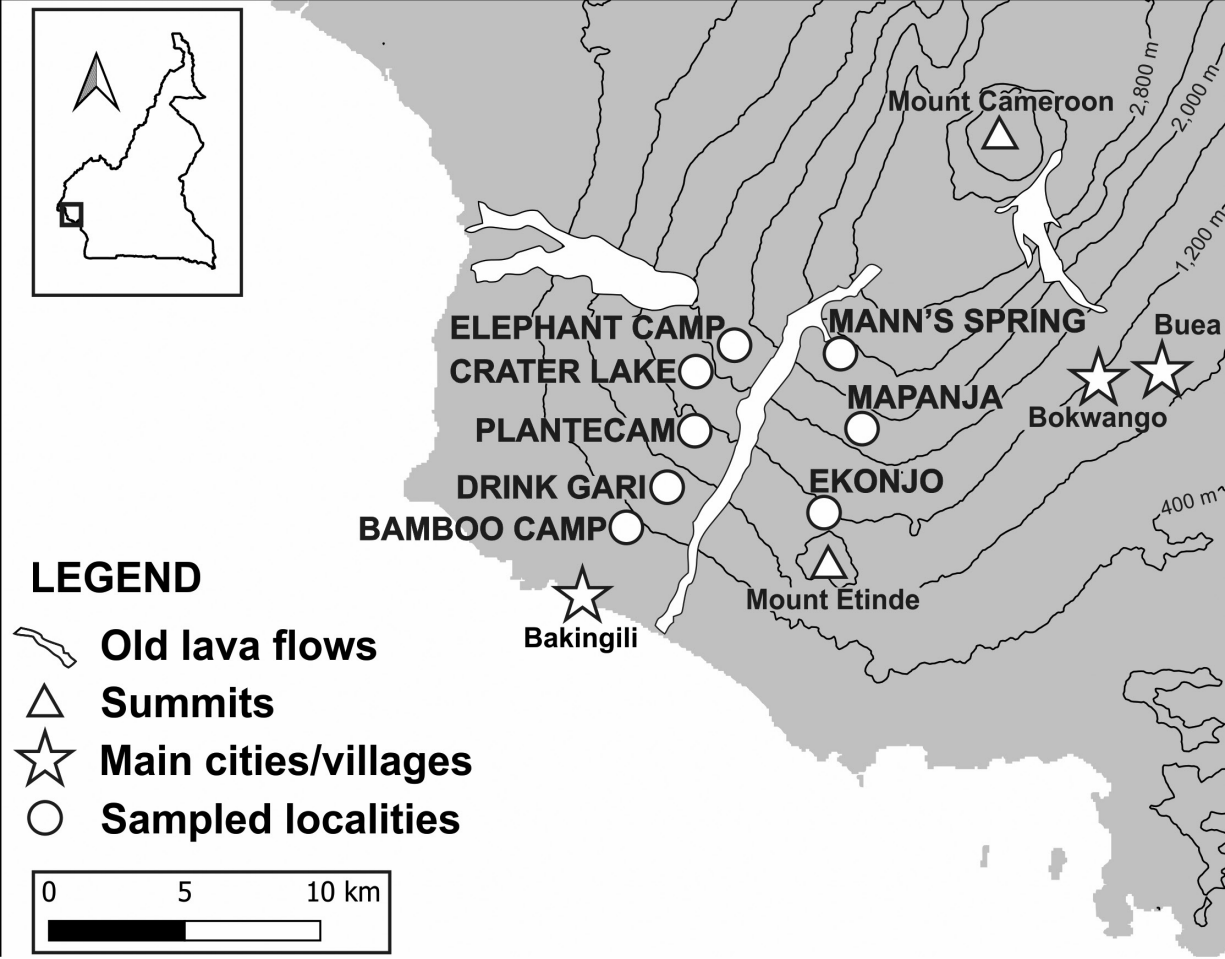
Map of the Mount Cameroon region with the sampling localities.

All specimens were collected in eight rainforest localities on the south-western and southern slopes of Mount Cameroon at elevations ranging from 350 to 2200 m a.s.l. between November 2014 and October 2017 (Fig. 1). All specimens were attracted to light. The detailed sampling protocol was described in Ustjuzhanin et al. (2018). Holotypes will be stored in NECJU, paratypes will be split between NECJU and CUK.

Genitalia of most specimens were dissected and examined. For their dissection and preparation of permanent slides, we followed a protocol described in Ustjuzhanin et al. (2018). Each permanent preparation received a unique code under which it is searchable in the collections where they are stored; the relevant numbers are listed in captions of the genitalia figures.

### Sampling localities

All sampling localities (Fig. 1) are listed below; the localities not included in Ustjuzhanin et al. (2018) are marked with *:

**Bamboo Camp.** Bamboo Camp (350 m a.s.l.), Mount Cameroon (SW slope), 4.0879°N, 9.0505°E; a lowland rainforest with historical disturbances from selective logging.

*Crater Lake. Crater Lake camp (1450 m a.s.l.), Mount Cameroon (SW slope), 4.1443°N, 9.0717°E; a submontane rainforest locally disturbed by forest elephants.

**Drink Gari.** Drink Gari camp (650 m a.s.l.; also known as “Drinking Gari”), Mount Cameroon (SW slope), 4.1014°N, 9.0610°E; a lowland rainforest with canopy layer presumed to be closed.

*Ekonjo. Ekonjo camp (1150 m a.s.l.), Mount Cameroon (S slope), 4.0881°N, 9.1168°E; an upland closed-canopy rainforest.

**Elephant Camp.** Elephant Camp (1850 m a.s.l.), Mount Cameroon (SW slope), 4.1170°N, 9.0729°E; a montane forest with a sparse canopy layer as a consequence of natural disturbances by forest elephants.

*Mann’s Spring. Mann’s Spring camp (2200 m a.s.l.), Mount Cameroon (SW slope), 4.1428°N, 9.1225°E; a montane forest at the natural timberline.

*Mapanja. Mapanja camp (1850 m a.s.l.), Mount Cameroon (S slope), 4.1157°N, 9.1315°E; a montane forest with canopy layer presumed to be closed.

**PlanteCam.** PlanteCam camp (1100 m a.s.l.; also misspelled as “Planticamp”), Mount Cameroon (SW slope), 4.1175°N, 9.0709°E; an upland rainforest in the transition between the lowland and montane zones, with a sparse canopy layer as a consequence of natural disturbances by forest elephants.

## Species descriptions

### Alucita bakweri

Taxon classificationAnimaliaLepidopteraAlucitidae

Ustjuzhanin & Kovtunovich sp. nov.

C69F508A-9011-56B1-B009-2FA0DA40023F

http://zoobank.org/4CCBE08C-2366-40FF-B52F-D7B3D60DF816

Figs 2 , 3

#### Type material.

***Holotype*** • 1 male, (NECJU 201901) Cameroon, PlanteCam, 1100 m a.s.l., Mount Cameroon (SW slope), 4.1175°N, 9.0709°E, 11–18.XII.2014, lgt. V. Maicher, Sz. Sáfián, Š. Janeček, R. Tropek.

#### Diagnosis.

In the yellowish colour of its wings, this species resembles *Alucita ferruginea* Walsingham, 1881, *Alucita balioxantha* (Meyrick, 1921), and *Alucita compsoxantha* (Meyrick, 1924), from which it differs in the structure of male genitalia. Genitalia of the new species differ from *A. balioxantha* by a thin sharp gnathos, prolonged saccus, and short, wide and wing-like valvae (Fig. 19). *Alucita balioxantha* gnathos is wider, with a blunt and round top, the saccus is archlike and not prolonged, and valvae are thin and lancet-like. In the shape of the uncus and gnathos of the male genitalia, *A. bakweri* is similar to *A. ferruginea*, from which it differs in having an elongated, narrowing saccus without the distinct notch on its outer edge. Additionally, unlike *A. ferruginea*, *A. bakweri* has clusters of small acicular cornuti apically on the aedaeagus. Moreover, its valvae are short, wide and wing-like, with a bundle of thin needle-like cornuti in its apical part. Whilst male genitalia of the new species have a forked uncus and comparatively short aedeagus, *A. compsoxantha* has a simple short uncus and a very long aedeagus.

#### External characters.

Wingspan 18 mm. Head with white scales, thorax and tegulae yellowish-brown. Labial palpus yellow, thin, straight, twice as long as longitudinal eye diameter. Third segment discrete, directed forward, median part framed by brown lines, apically pale and sharp. Antenna white, basally thickened. Wings yellowish-brown, with four white transverse lines. Wings basally darkened with brown scales. Abdomen yellowish-brown. Hind leg pale yellow.

#### Male genitalia.

Uncus long, distally widened, apically with triangle notch. Gnathos narrow, apically tapered, in length equal to uncus. Gnathos arms short, thick, smoothly bent inwards. Valves short, wide, wing-shaped, apically with bundle of fine acicular setae. Anellus arms wide, short. Saccus elongated, narrow triangular. Aedeagus almost straight, with an elongated uncinate cornutus in middle, groups of fine acicular cornuti distally, and narrow sharp cornuti sticking out apically.

**Figures 2, 3.**
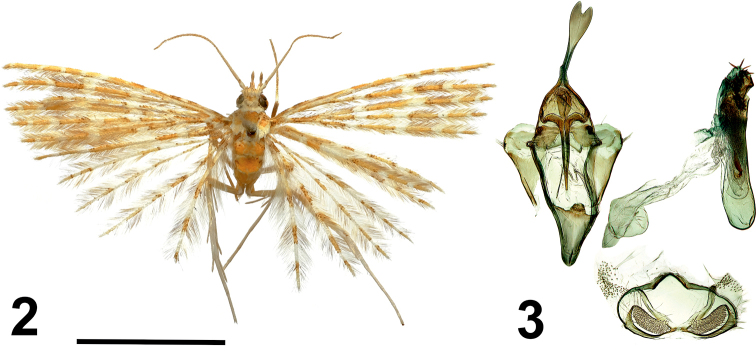
*Alucita bakweri* Ustjuzhanin & Kovtunovich, sp. nov. **2** adult male, Holotype, NEJCU **3** male genitalia, Holotype, preparation slide no. 201901. Scale bar: 5 mm.

#### Distribution.

Cameroon.

#### Flight period.

December.

#### Etymology.

We name the species after the Bakweri people, the main ethnic group of the Mount Cameroon region. Without the priceless assistance of numerous local people our project would not be possible. We hope such dedication will encourage protection of the species’ habitats.

### Alucita jana

Taxon classificationAnimaliaLepidopteraAlucitidae

Ustjuzhanin & Kovtunovich sp. nov.

EA260D14-8732-5B50-868E-42DA5BE03501

http://zoobank.org/2EE6AAB9-8CCB-4123-B957-786B6776CB92

Figs 4 , 5

#### Type material.

***Holotype*** • male, (NECJU 201902) Cameroon, PlanteCam, 1100 m a.s.l., Mount Cameroon (SW slope), 4.1175°N, 9.0709°E, 09–14.IV.2015, lgt. V. Maicher, Sz. Sáfián, S. Janeček, R. Tropek. ***Paratypes*** • 1 male, (CUK), Ekonjo, 1150 m a.s.l., Mount Cameroon (SW slope), 4.0881°N, 9.1168°E, 24.X.2017, lgt.V. Maicher, P. Potocký, S. Delabye • 1 male, (CUK), Mapanja camp, 1850 m a.s.l., Mount Cameroon (SW slope), 4.1157°N, 9.1315°E, 25.X.2017, V. Maicher, P. Potocký, S. Delabye.

#### Diagnosis.

The new species is similar in the shape of the uncus and valves of the male genitalia to *Alucita tesserata* (Meyrick, 1918) (Fig. 20), but it differs in the apically narrow gnathos and the presence of a group of fine acicular cornuti in the aedeagus. *Alucita jana* also differs from *A. tesserata* in the wing colouration.

#### External characters.

Wingspan 15 mm. Head, thorax and tegulae with brown appressed scales. Labial palpus wide, short, 1.5 longer than longitudinal eye diameter, slightly bent upwards, brown scaled inside and outside. Third segment discrete, apically sharp. Antenna yellowish-brown. Wings yellowish-brown, distinctive pale brown band medially. Forewings show a dark brown postmedial band. Forewing basally with dark brown scales, hindwing basally light. Distally, alternating portions of brown and yellowish scales. Fringe of wings yellow, with alternating portions of brown hairs. Hind leg pale yellow.

#### Male genitalia.

Uncus long, apically slightly widened. Gnathos long, distally widened, apically slightly tapered, equal to uncus in length. Valve short, finger-like, membranous. Anellus arms narrow, long, apically slightly widened. Saccus with oval outer edge. Aedeagus straight, basally widened, apically with a group of fine acicular cornuti.

**Figures 4, 5.**
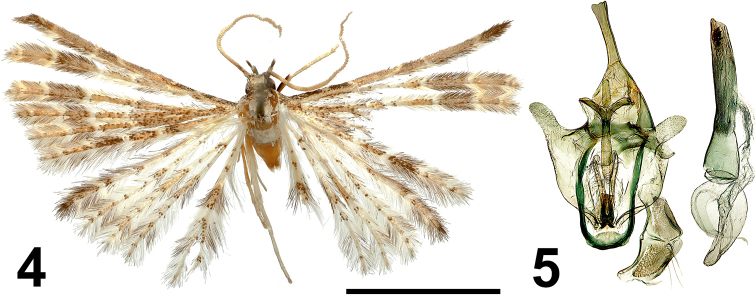
*Alucita jana* Ustjuzhanin & Kovtunovich, sp. nov. **4** adult male, Holotype, NEJCU **5** male genitalia, Holotype, preparation slide no. 201902. Scale bar: 5 mm.

#### Distribution.

Cameroon.

#### Flight period.

April, October.

#### Etymology.

The species name is a noun in apposition in honour to Robert Tropek’s mother, Jana Tropková.

### Alucita bakingili

Taxon classificationAnimaliaLepidopteraAlucitidae

Ustjuzhanin & Kovtunovich sp. nov.

29B9D97F-9A18-5C0A-A65A-8DFA56DDF31B

http://zoobank.org/C1503460-2901-4437-BFA7-49D660E2462F

Figs 6–8

#### Type material.

***Holotype*** • male (NECJU 201903) Cameroon, Bamboo Camp, 350 m a.s.l., Mount Cameroon (SW slope), 4.0879°N, 9.0505°E, 12–20.XII.2014, lgt. V. Maicher, Sz. Sáfián, S. Janeček, R. Tropek. ***Paratypes*** • 1 male (CUK), same data as the holotype • 1 female (NECJU 201908), same data as the holotype • 1 male, (CUK), PlanteCam, 1100 m a.s.l., Mount Cameroon (SW slope), 4.1175°N, 9.0709°E, 29.I.-07.II.2016, lgt. V. Maicher, Sz. Sáfián, R. Tropek.

#### Diagnosis.

The new species resembles *Alucita fokami* Ustjuzhanin & Kovtunovich, 2018 in external appearance but it substantially differs in both male and female genitalia (for genitalia of *A. fokami*, see Ustjuzhanin et al. 2018). *Alucita bakingili* is similar to *Alucita seychellensis* (T.B. Fletcher, 1910) in male genitalia (illustrated in Ustjuzhanin and Kovtunovich 2016), specifically in the sclerotized process on the sacculus. *Alucita bakingili* also differs from *A. seychellensis* in its wide gnathos, the narrow triangular valves and the short narrow uncus. In the female genitalia, the new species is similar to *Alucita rhaptica* (Meyrick, 1920) (Fig. 21), from which it differs in its rectangular lamina postvaginalis and in the longer and narrower ductus.

#### External characters.

Wingspan 12–15 mm, holotype 12 mm. Head, thorax and tegulae with dark grey scales and an admixture of white scales. Labial palpus grey outside, white inside, 1.5 times longer than longitudinal eye diameter, directed forward. Third segment short, apically slightly sharpened. Antenna pale grey, distinct dark elongated spot basally just beyond scape. Wings mottled, yellowish-grey, medially with a poorly expressed yellowish-brown band. Alternating portions of grey and white scales shaped as elongated strokes, spots and points on lobe of all wings. Fringe with alternating portions of grey and white hairs. Hind leg pale yellow.

#### Male genitalia.

Uncus short, straight, slightly widened apically. Gnathos wide, sharply narrowing apically, a little longer than uncus. Valve short, narrow triangular, membranous. Sacculus with membranous process containing a large sclerotized uncinate process in lower part. Anellus arms very long, basally wide, medially narrowing, apically widened and slightly bent. Saccus with oval outer edge. Aedeagus long, straight, with two robust cornute in this medial part.

#### Female genitalia.

Papilla analis narrow, elongated. Posterior apophyses thin, straight. Anterior apophyses also thin, straight, equal in length to posterior apophyses. Lamina postvaginalis sclerotized, wide, rectangular, with blunt angular lateral projections. Antrum corrugated, wide, short. Ductus wide, medially swollen. Ductus seminalis short, well expressed. Bursa copulatrix small, oval, with robust elongated comb-shaped signum located basally and reaching base of ductus seminalis.

**Figures 6–8.**
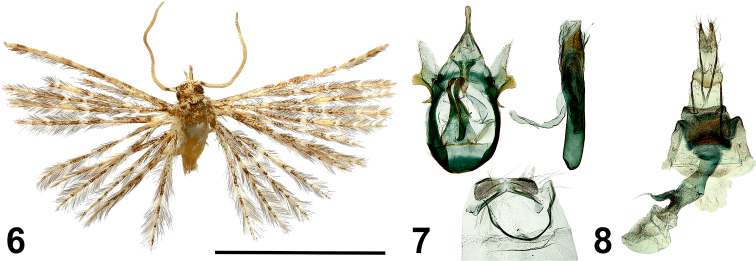
*Alucita bakingili* Ustjuzhanin & Kovtunovich, sp. nov. **6** adult male, Holotype, NEJCU **7** male genitalia, Holotype, preparation slide no. 201903 **8** female genitalia, Paratype, preparation slide no. 201908. Scale bar: 5 mm.

#### Distribution.

Cameroon.

#### Flight period.

December to February.

#### Etymology.

The species is named after Bakingili, a village at the southern foothills of Mount Cameroon, in which area it was collected. Numerous people from the village helped our project as field assistants and by many other ways, and therefore the community was crucial for its success. The Bakingili people are also necessary for protection of the species’ natural area.

### Alucita tatjana

Taxon classificationAnimaliaLepidopteraAlucitidae

Ustjuzhanin & Kovtunovich sp. nov.

721E1EED-8A3B-50CE-8B9C-A6554BB72D8C

http://zoobank.org/2CCDE55D-7D9C-413C-A5B0-42833DE8F770

Figs 9 , 10

#### Type material.

***Holotype*** • female, (NECJU 201904) Cameroon, PlanteCam, 1100 m a.s.l., Mount Cameroon (SW slope), 4.1175°N, 9.0709°E, 09–14.IV.2015, V. Maicher, Sz. Sáfián, S. Janeček, R. Tropek; ***Paratype*** • 1 female, (CUK), Ekonjo, 1150 m a.s.l., Mount Cameroon (S slope), 4.0881°N, 9.1168°E, 24.X.2017, lgt. V. Maicher, P. Potocký, S. Delabye.

#### Diagnosis.

The new species resembles *Alucita mischenini* Ustjuzhanin & Kovtunovich, 2018 in the light colour of its wings and the wide medial band, but it differs in its larger size (21 mm vs. 12–15 mm wingspan). Additionally, the position and shape of dark markings in the basal and distal portions of the wings differentiates *A. tatjana* from *A. mischenini* whose forewings bear extensive brown areas in in the basal portions and almost continuous dark brown marks covering the distal halves of the first two forewing lobes (Ustjuzhanin et al. 2018). Additionally, forewings of the new species differ from *A. mischenini* by the continuation of the brown medial band through most of the length of the second forewing lobe. In female genitalia, *A. tatjana* is similar to *A. compsoxantha* Meyrick, 1924 (Fig. 22), especially in the shape of the antrum and the absence of any signa in the bursa copulatrix. However, *A. tatjana* differs in its shorter and wider ductus and substantially shorter anterior and posterior apophyses. These species also differ in the colouration of their wings.

#### External characters.

**Figures 9, 10.**
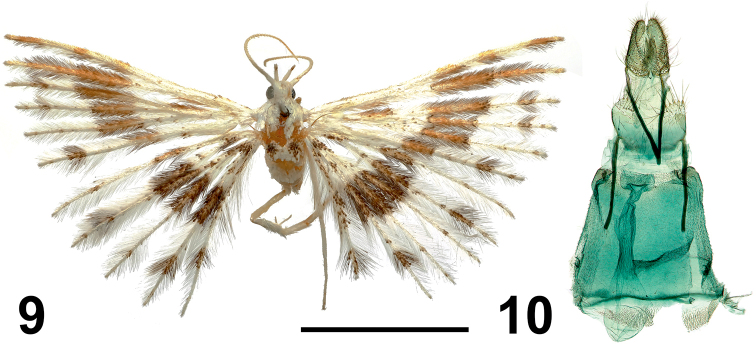
*Alucita tatjana* Ustjuzhanin & Kovtunovich, sp. nov. **9** adult female, Holotype, NEJCU **10** female genitalia, Holotype, preparation slide no. 201904. Scale bar: 5 mm.

Wingspan 21 mm. Head, thorax and tegulae with appressed white scales. Labial palpus twice as long as longitudinal eye diameter, white, with a brown band of the third segment, thin, apically tapered, sharp. Antenna white. Scape extended and flattened. Wings white with well-expressed wide median band, brown on forewing and almost black on hindwing. Subterminal band narrower, consisting of brown spots of scales, intermittently traced throughout all lobes. First forewing lobe apically brown. Second forewing lobe with brown colouration continuous between median band and subterminal band. Forewing basally white with well-expressed dark brown fringes near the base of the cleft between the second and third lobes. Hindwing basally white with scattered dark brown scales and a prominent dark brown marking across base of second to sixth lobes. Hind leg pale yellow (although not so apparent in Fig. 9).

#### Female genitalia.

Papilla analis narrow, elongated. Posterior and anterior apophyses thick, straight. Anterior apophyses equal in length to posterior apophyses. Antrum wide, goblet-like. Ductus short, expanded in median part around junction with ductus seminalis, and corrugated at junction with bursa copulatrix. Bursa copulatrix small, oval, without signa.

#### Distribution.

Cameroon.

#### Flight period.

April, October.

#### Etymology.

The species is a noun in apposition in honour to Petr Ustjuzhanin’s sister, Tatjana Ustjuzhanina.

### Alucita zuza

Taxon classificationAnimaliaLepidopteraAlucitidae

Ustjuzhanin & Kovtunovich sp. nov.

FB270130-C9C9-542A-B5AB-0D8D4232D724

http://zoobank.org/3EFBC3D5-E44D-4F58-A1D4-3E5D049D58E3

Figs 11 , 12

#### Type material.

***Holotype*** • 1 male, (NECJU 201905) Cameroon, Drink Gari, 650 m a.s.l., Mount Cameroon (SW slope), 4.1014°N, 9.0610°E, 11–23.IV.2015, lgt. V. Maicher, Sz. Sáfián, Š. Janeček, R. Tropek; ***Paratypes*** • 1 male, (CUK), same data as the holotype • 1 male (CUK), PlanteCam, 1100 m a.s.l., Mount Cameroon (SW slope), 4.1175°N, 9.0709°E, 29.I–07.II.2016, lgt. V. Maicher, Sz. Sáfián, R. Tropek • 1 male (CUK), Drink Gari, 650 m a.s.l., Mount Cameroon (SW slope), 4.1014°N, 9.061°E, 20.XI–10.XII.2014, lgt. V. Maicher, Sz. Sáfián, R. Tropek.

#### Diagnosis.

In its male genitalia, the new species shares the elongated saccus and absence of valves with *A. fokami* (illustrated in Ustjuzhanin et al. 2018), but it differs in the narrow uncus which is not expanded apically, and in the absence of a long needle-like cornutus in the aedeagus.

#### External characters.

Wingspan 12 mm. Head white, with two brown spots between antennae. Thorax and tegulae white. Labial palpus straight, 1.5 times as long as longitudinal eye diameter, with alternating white and dark bands on each segment. Third segment short, not tapered apically. Antenna yellow, with small dark brown spots basally, just above scape. Wings mottled in their dark parts. Forewing darker than hindwing, with predominance of dark brown spots and strokes, while these spots and strokes are less expressed on the hindwing, with predominance of pale-yellow portions. Wings basally white, although locally darkened with dark brown scales. Fringe with alternating portions of light and brown hairs. Hind leg pale yellow.

#### Male genitalia.

Uncus straight, finger-like, of even width. Gnathos and its arms wide, short. Valves absent. Anellus arms short, narrow, arched, apically sharp. Saccus very long, narrow, elongated, slightly expanded medially. Aedeagus straight, medially with two small, spinous cornuti (not apparent on Fig. 12).

**Figures 11, 12.**
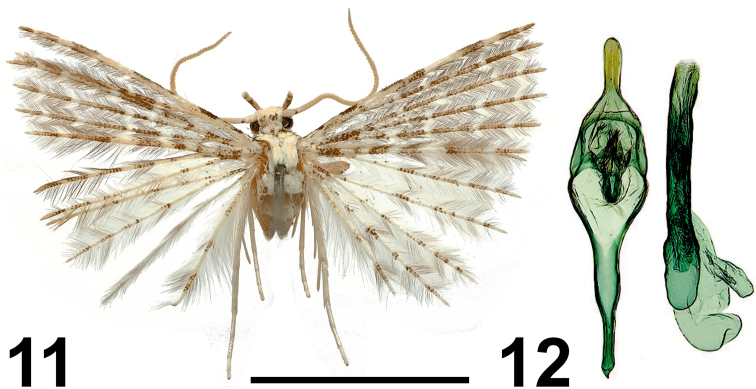
*Alucita zuza* Ustjuzhanin & Kovtunovich, sp. nov. **11** adult male, Holotype, NEJCU **12** male genitalia, Holotype, preparation slide no. 201905. Scale bar: 5 mm.

#### Distribution.

Cameroon.

#### Flight period.

November till February, April.

#### Etymology.

The species name is a noun in apposition. It was named in honour of the ichthyologist Zuzana Musilová, Robert Tropek’s wife.

### Alucita deja

Taxon classificationAnimaliaLepidopteraAlucitidae

Ustjuzhanin & Kovtunovich sp. nov.

0855E441-77EB-52E9-9FA7-BB614E362120

http://zoobank.org/9C094272-0A16-4CEA-AB05-CD4E3C04D27B

Figs 13–15

#### Type material.

***Holotype*** • male (NECJU 201906), Cameroon, Bamboo Camp, 350 m a.s.l., Mount Cameroon (SW slope), 4.0879°N, 9.0505°E, 20.XII.2014, lgt. V. Maicher, Sz. Sáfián, Š. Janeček, R. Tropek. ***Paratypes*** • 1 female (CUK), PlanteCam, 1100 m a.s.l., Mount Cameroon (SW slope), 4.1175°N, 9.0709°E, 11–18.XII.2014, lgt. V. Maicher, Sz. Sáfián, Š. Janeček, R. Tropek • 1 female (NECJU 201909), PlanteCam, 1100 m a.s.l., Mount Cameroon (SW slope), 4.1175°N, 9.0709°E, 11–23.IV.2015, lgt. V. Maicher, Sz. Sáfián, Š. Janeček, R. Tropek.

#### Diagnosis.

*Alucita deja* differs from other *Alucita* species by the distinctive white mirrors on its abdomen. The new species’ male genitalia are similar to *A. janeceki* Ustjuzhanin & Kovtunovich, 2018 in the absence of valves and the shape of the saccus (illustrated in Ustjuzhanin et al. 2018), but *A. deja* differs in the smoothly rounded apex of its uncus, the apically sharp anellus arms, jagged acicular cornuti in the aedeagus, and the wing colouration.

#### External characters.

Female wingspan 15 mm, male wingspan 13 mm. Head with pure-white appressed scales. Thorax and tegulae also pure white anteriorly, sharply contrasting with dark brown posterior portions. Labial palpus short, 1.5 times as long as longitudinal eye diameter. Third segment short, not tapered apically. Antenna brown. Scape pure white. Wings and fringes dark brown. First lobe of forewing with white band around 1/3, narrow white band around 3/5 and yellowish-brown band edged with white around 4/5. Several irregular fine white cross-lines across all other lobes and fringes of both wings. Hind leg yellowish-brown. Fourth, sixth and last tergites of abdomen with mirrors of pure-white scales. Abdomen completely pure-white ventrally.

#### Male genitalia.

Uncus straight, long, of even width. Gnathos short, wide, apically expanded. Valves not developed. Anellus arms straight, apically sharp. Saccus shaped as elongated oval, with small notch at apex. Aedeagus robust, slightly shorter than entire genital structure, with jagged acicular cornuti from its medial part to apex.

#### Female genitalia.

Papilla analis narrow, elongated. Posterior apophyses thin, straight, shorter than anterior apophyses. Antrum wide, sclerotized, shaped as truncated tube with extended ostium. Outer edge of ostium jagged. Ductus wide and corrugated at its confluence to antrum and bursa copulatrix. Ductus seminalis short, bag-like, membranous, departing from confluence of ductus to antrum. Bursa copulatrix of irregular oval shape with elongated protrusion at apex.

**Figures 13–15.**
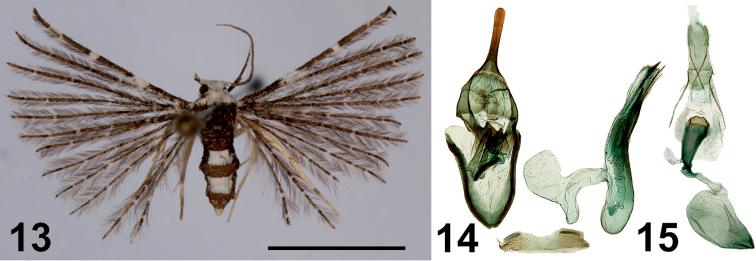
*Alucita deja* Ustjuzhanin & Kovtunovich, sp. nov. **13** adult female, Paratype, NEJCU **14** male genitalia, Holotype, preparation slide no. 201906 **15** female genitalia, Paratype, preparation slide no. 201909. Scale bar: 5 mm.

#### Distribution.

Cameroon.

#### Flight period.

April, December.

#### Etymology.

The species name is a noun in apposition in honour of the limnologist and cyanobacteria specialist, Andreja Kust, a dear soulmate of Vincent Maicher.

### Alucita bokwango

Taxon classificationAnimaliaLepidopteraAlucitidae

Ustjuzhanin & Kovtunovich sp. nov.

3B9B5C2B-8144-52F4-B6E3-8BB986D38F80

http://zoobank.org/C84BA8AD-3588-494F-86C8-55DEA44AC841

Figs 16–18

#### Type material.

***Holotype*** • male (NECJU 201907), Cameroon, Elephant Camp, 1850 m a.s.l., Mount Cameroon (SW slope), 4.1170°N, 9.0729°E, 19–24.XI.2014, lgt. V. Maicher, Sz. Sáfián, Š. Janeček, R. Tropek. ***Paratypes*** • 1 female, (NECJU 201910), + 16 ex., (NECJU, CUK), same data as holotype • 1 male, (CUK), PlanteCam, 1100 m a.s.l., Mount Cameroon (SW slope), 4.1175°N, 9.0709°E, 06–15.II.2016, lgt. V. Maicher, Sz. Sáfián, R. Tropek • 2 ex., (CUK, NECJU), PlanteCam, 1100 m a.s.l., 4.1175°N, 9.0709°E, 11–18.XII.2014, lgt. V. Maicher, Sz. Sáfián, Š. Janeček, R. Tropek • 1 male (CUK), Crater Lake, 1500 m a.s.l., Mount Cameroon (SW slope), 4.1443°N, 9.0717°E, 11–21.II.2016, lgt. P. Potocký, Sz. Sáfián, J. Mertens, Š. Janeček, R. Tropek • 1 male (CUK), Elephant Camp, 1850 m a.s.l., Mount Cameroon (SW slope), 4.1170°N, 9.0729°E, 17–22.II.2017, lgt. P. Potocký, Sz. Sáfián, J. Mertens, Š. Janeček, R. Tropek • 1 male (NECJU), Mann’s Spring, 2200 m a.s.l., Mount Cameroon (SW slope), 4.1428°N, 9.1225°E, 16–21.IV.2017, lgt. V. Maicher, P. Potocký, S. Delabye • 1 male (CUK), Crater Lake, 1500 m a.s.l., Mount Cameroon (SW slope), 4.1443°N, 9.0717°E, 23–29.IV.2017, lgt. V. Maicher, P. Potocký, S. Delabye • 8 ex., (NECJU, CUK), Elephant Camp, 1850 m a.s.l., Mount Cameroon (SW slope), 4.1170°N, 9.0729°E, 18–26.IV.2017, lgt. V. Maicher, P. Potocký, S. Delabye • 5 ex. (CUK, NECJU), Mapanja, 1850 m a.s.l., Mount Cameroon (S slope), 4.1157°N, 9.1315°E, 05–14.V.2017, lgt. V. Maicher, P. Potocký, S. Delabye • 6 ex. (CUK, NECJU), Mapanja, 1850 m a.s.l., Mount Cameroon (SW slope), 4.1157°N, 9.1315°E, 23–28.X.2017, lgt. V. Maicher, P. Potocký, S. Delabye.

#### Diagnosis.

The new species resembles *Alucita chloracta* (Meyrick, 1908) in its external characters and the structure of the male genitalia. Genitalia of *A. bokwango* differs from *A. chloracta* in the needle-like apex of the uncus and the oval apical expansions of the valvae, whilst the uncus of *A. chloracta* is noticeably widened apically with a notch and valvae have a rounded apex. In its female genitalia, *A. bokwango* differs from *A. chloracta* (Fig. 24) in the short narrow ductus, wide funnel-shaped antrum and absence of a plaque-like signum in the bursa copulatrix. The wing colouration of *A. bokwango* is more contrasted than in *A. chloracta* (Fig. 23), and the new species is also substantially larger (23–25 mm vs. 15–16 mm wingspan).

#### External characters.

Wingspan 23–25 mm. Head, thorax, tegulae and abdomen all dark brown. Labial palpus yellowish-brown, three times as long as longitudinal eye diameter, directed forward. Third segment thin, apically tapered. Antenna pale brown, with small dark brown spots basally, just above scape. Wings brown, outer edge slightly lighter. Medial band on all wings whitish, almost transparent, interspersed with brown portions of hairs on lobes fringe. Hind leg yellow.

#### Male genitalia.

Uncus thin, long, needle-shaped. Gnathos equal to uncus in length, apically sharp. Valves long, membranous, smoothly forming an oval apically. Anellus arms straight, narrow, equal to gnathos in their length. Saccus oval. Aedeagus robust, thick, almost equal in length to genital structure without uncus, two spinous cornuti distally.

#### Female genitalia.

Papilla analis oval, wide throughout length. Posterior apophyses slightly shorter than anterior, thick and slightly undulated. Antrum wide, funnel-like. Ductus short, thin, membranous. Bursa copulatrix large, oval, with two large, lanceolate signa (although not clearly apparent in Fig. 18).

**Figures 16–18.**
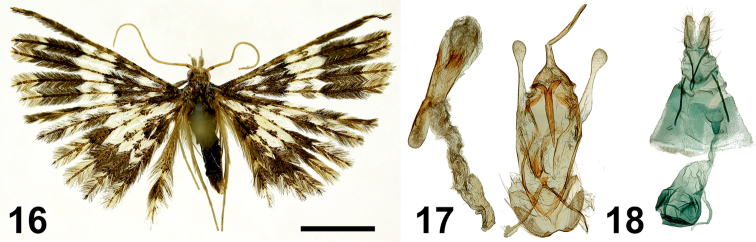
*Alucita bokwango* Ustjuzhanin & Kovtunovich, sp. nov. **16** adult male, Paratype, NEJCU **17** male genitalia, Holotype, preparation slide no. 201907 **18** female genitalia, Paratype, preparation slide no. 201910. Scale bar: 5 mm.

#### Distribution.

Cameroon.

#### Flight period.

October-May

#### Etymology.

The species is named after Bokwango, a village on the eastern slope of Mount Cameroon where our project established its main base and where we made a lot of good friends. Many of the species records were also made in forests belonging to the village. Last but not least, most of our field assistants and other helpers came from Bokwango and we are thankful to all of them for the success of the project. We strongly believe this dedication will also help protect the unique biodiversity of the region.

#### Note.

18 specimens from Elephant Camp, 19–24.XI.2014, and two specimens from PlanteCam, 11–18.XII.2014, (all lgt. V. Maicher, Sz. Sáfián, Š. Janeček, R. Tropek), were erroneously indicated as *A. chloracta* by Ustjuzhanin et al. (2018). The other *A. chloracta* specimens referred by Ustjuzhanin et al. (2018) from Bamboo Camp, Drink Gari and PlanteCam were identified correctly.

**Figures 19–24.**
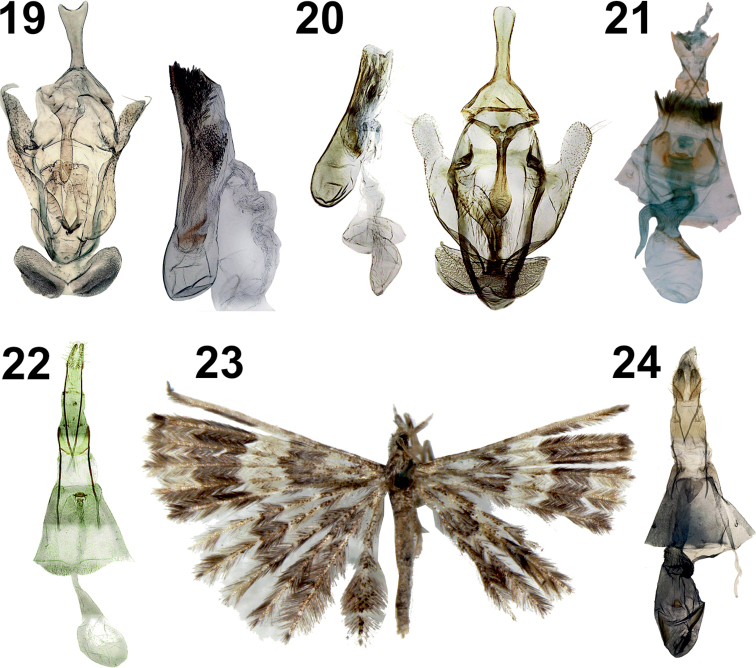
Comparative illustrations of other *Alucita* species mentioned in the new species’ diagnoses **19***A. balioxantha* (Meyrick, 1921), type, male genitalia **20***A. tesserata* (Meyrick, 1918), type, male genitalia **21***A. rhaptica* (Meyrick, 1920), female genitalia **22***A. compsoxantha* (Meyrick, 1924), type, female genitalia **23***A. chloracta* (Meyrick, 1908), type, adult female **24***A. chloracta*, type, female genitalia.

## Discussion

With the seven newly described *Alucita* species, the known diversity of many-plumed moths on Mount Cameroon has been increased to 22 species (Table 1). Altogether, 19 of these species were collected in the single locality, PlanteCam (Table 1), but the general diversity patterns will be analysed only once the complete *Alucita* material from our collections is processed. This comprises more than a quarter of the known Afrotropical diversity of many-plumed moths. At most, only a few species of the group are known from other localities in the region (Ustjuzhanin et al. 2018; De Prins and De Prins 2019). Although microlepidopteran diversity in the Guineo-Congolian forest zone remains largely unknown, discoveries of multiple undescribed species of many-plumed moths from a single locality is unexpected (Ustjuzhanin et al. 2018).

**Table 1.** Summary of all specimens of *Alucita* moths sampled on Mount Cameroon in this study and by Ustjuzhanin et al. (2018).

Sampling locality	Altitude	*A. acalyptra*	*A. bakingili*	*A. bakweri*	*A. besongi*	*A. bokwango*	*A. chloracta*	*A. coffeina*	*A. deja*	*A. escobari*	*A. fokami*	*A. jana*	*A. janeceki*	*A. lidiya*	*A. longipenis*	*A. ludmila*	*A. megaphimus*	*A. mischenini*	*A. olga*	*A. spicifera*	*A. tatjana*	*A. zinovievi*	*A. zuza*
Bimbia-Bonadikombo	30 m																	1					
Bamboo Camp	350 m	1	3		3		7		1	2	2		2	1		2	6		4				
Drink Gari	650 m						1				1		1	1		1	3						3
PlanteCam	1100 m		1	1		3	3	1	2	2	1	1		2	1	1	5	2	1	1	1	1	1
Ekonjo	1150 m											1									1		
Crater Lake	1450 m					2																	
Elephant Camp	1850 m					27									7					7			
Mapanja	1850 m					11						1											
Mann’s Spring	2200 m					1																	

Mount Cameroon is known to harbour high diversity in many groups, including Lepidoptera (e.g., Ballesteros-Mejia et al. 2013; Maicher et al. 2016; Przybyłowicz et al. 2019; Delabye et al. 2020). This is usually explained as a result of the combination of its position at the border between the Guinean and Congolian biogeographic regions, its diversity of habitats along the elevational and precipitation gradients, and its isolated “sky island” character (Ustjuzhanin et al. 2018). Nevertheless, even such unique combination of conditions can hardly explain why Mount Cameroon so strongly outnumbers all other Afrotropical localities in the species richness of its many-plumed moths. Of the known 22 *Alucita* species, 16 have been described from the site and have not yet been found anywhere else. Although it is highly expected that some of these will be distributed more widely, several of the newly described species are distinctive and unmistakable in their appearance. These are unlikely to have been overlooked in collections. Therefore, we expect that most of this many-plumed moth diversity is endemic to the study area. Several other potentially endemic species of moths (e.g., Yakovlev and Sáfián 2016; De Prins and De Prins 2019; Przybyłowicz et al. 2019;) and butterflies (e.g., Larsen 2005; Sáfián and Tropek, 2016; Sáfián et al. 2019) are already known from Mount Cameroon. Nevertheless, the real character of the endemism within Alucitidae on Mount Cameroon, as well as the mechanisms underlying the group’s speciation (or even local radiation), will need more detailed research.

## Supplementary Material

XML Treatment for Alucita bakweri

XML Treatment for Alucita jana

XML Treatment for Alucita bakingili

XML Treatment for Alucita tatjana

XML Treatment for Alucita zuza

XML Treatment for Alucita deja

XML Treatment for Alucita bokwango
